# Milky spots: omental functional units and hotbeds for peritoneal cancer metastasis

**DOI:** 10.1007/s13277-016-4887-3

**Published:** 2016-01-29

**Authors:** Jiuyang Liu, Xiafei Geng, Yan Li

**Affiliations:** 1 Department of Oncology, Hubei Key Laboratory of Tumor Biological Behaviors and Hubei Cancer Clinical Study Center, Zhongnan Hospital of Wuhan University, No. 169 Donghu Road, Wuchang District, Wuhan, 430071 China; 2 Department of Peritoneal Cancer Surgery, Beijing Shijitan Hospital Affiliated to the Capital Medical University, Tieyilu 10, Yangfangdian, Haidian District, Beijing, 100038 China

**Keywords:** Omental milky spots, Cancer, Invasion and metastasis, Peritoneal carcinomatosis

## Abstract

As the most common metastatic disease of abdomen pelvic cavity cancer, peritoneal carcinomatosis (PC) renders significant negative impact on patient survival and quality of life. Invasive peritoneal exfoliated cancer cells (PECCs) preferentially select the omentum as a predominant target site for cancer cell colonization and proliferation compared with other tissues in the abdominal cavity. The precise pathogenic mechanism remains to be determined. As omental milky spots (MSs) are the major implantation site for malignant cells in peritoneal dissemination, researches on mechanisms of PC have been mainly focused on MS, primitive lymphoid tissues with unique structural features, and functional characteristics. To date, extensive biophysical and biochemical methods have been manipulated to investigate the MS exact function in the peritoneal cavity. This review summarized MS as hotbeds for PECC. The anatomical distribution was briefly described first. Then, MS histology was systematically reviewed, including morphological features, cellular constituents, and histological staining methods. At last, the roles of MS in PC pathological process were summarized with special emphasis on the distinct roles of macrophages.

## Introduction

Tumor invasion and metastasis remain the lethal causes of death and great challenges for cancer patients even after multimodality clinical treatments. As a regional tumor progression in abdomen pelvic cavities, peritoneal carcinomatosis (PC) mostly results from carcinomas of the stomach, colorectum, and ovary [1–5]. Characterized by the implantation of tumor nodules throughout the peritoneal cavity, PC has significant negative impacts on patient prognosis because of refractory ascites, intractable abdominal pain, and progressive intestinal obstruction. In gastric cancer (GC), almost 60 % of all causes of GC death is due to PC [6].

Different from hematogenous or lymphatic metastases, the pathological process of PC involves the following aspects of peritoneal exfoliated cancer cells (PECCs): adhesion, degradation, migration, angiogenesis, and immune evasion [7–9], in which gene modification and abundant bioactive compounds also participate. The precise pathophysiological mechanism remains unclear. However, increasing studies on PC have reached a consensus that PECCs specifically choose the omentum as the implantation site [10–13]. Macroscopically, the anatomical particularity of omentum consists in its positional adjacency to abdominal primary tumors. As the central regulator of peritoneal homeostasis, the omentum also performs active functions in regulating fluid and solute transport and promoting angiogenesis [14]. Once PC occurs within the omentum, malignant cells transported by peritoneal fluids tend to colonize and proliferate these surrounding neo-vessels. It has been well recognized that tumor invasion and metastasis are closely associated with the coevolution of cancer cells and tumor microenvironment [15–17]. On the basis of defined “invasion unit” by Fang et al. [18], the “pulse-mode” of cancer invasion and metastasis provides a new insight for cancer researches. Similarly in the omentum, there are numerous tiny functional units called milky spots (MSs), serving as suitable microenvironment for PECC [10, 19].

MSs were firstly observed as dense corpuscles resembling cotton wool in the omentum and pleura of rabbits in 1863 by Recklinghausen [20]. In 1874, Ranvier confirmed this discovery and named these corpuscles milky spots [21]. Since Hagiwara reported omental MS as the major implantation site for malignant cells in peritoneal dissemination in mice in 1993 [10], researchers have gradually noticed the relevance between MS and PC. Due to the difficulty of locating human MS with the naked eye, few researches had been dedicated to them until the 1990s. In the beginning, some researchers considered MS as lymphatic structures [22], whereas others regarded them as suppliers of peritoneal macrophages [23, 24]. In 2009, Rangel-Moreno et al. [25] provided compelling data to confirm MS as unique secondary lymphoid organs, which was also reported soon afterwards by Mebius [26]. To date, extensive biophysical and biochemical methods have been manipulated to detect and unravel MS unique structural features and functional characteristics. On the basis of previous work, the anatomical distribution, histological features, and roles in PC progression of MS have been systematically summarized in this review.

## Anatomical distribution of MS

Pleural and peritoneal cavities are repositories of MS [27]. In pleural cavity, MSs seem to be located mainly in the pleural fold behind the pericardium rather than lung parenchyma [27]. However, MS can be found in many tissues in abdomen pelvic cavities. Using P388 leukemia cells labeled with bromodeoxyuridine for i.p. injection of mice, Hagiwara et al. [10] established the descending order of tissue MS content as omentum, gonadal fat, mesenterium, posterior abdominal wall, stomach, liver, intestine, anterior abdominal wall, and lung. Except for it, there are still no straightforward researches on quantitative comparisons of pleural and peritoneal MS.

Subsequent studies applying peritoneal metastatic models also revealed that the omentum is a predominant site [12, 13, 28–30] for MS distribution. Additionally, Sorensen et al. [12] uniquely utilized an in vivo imaging system (IVIS) for closer microscopic analysis of green fluorescent protein (GFP) expressing B16 tumor cells injected i.p. and found that the tumor foci were localized to distinct areas coinciding with MS zones on the omentum.

In addition, Imai et al. [31] classified MS as type I (farther type) and type II (closer type) based on MS location relationship with blood vessels. However, recent studies using whole mount technique of the omentum had found that MS tended to distribute along a dense network of blood vessels [30].

## Histological features of MS

### Gross morphology and size

MSs with opaque patch shape [32] are similar in morphology (round or oval), while different in sizes, with diameters ranging from 349 to 756 μm [33]. MS densities decrease with age in the human omentum [32, 33], ranging from 40–50/cm^2^ in neonates to less than 10/cm^2^ in humans 70 years of age.

**Fig. 1:**
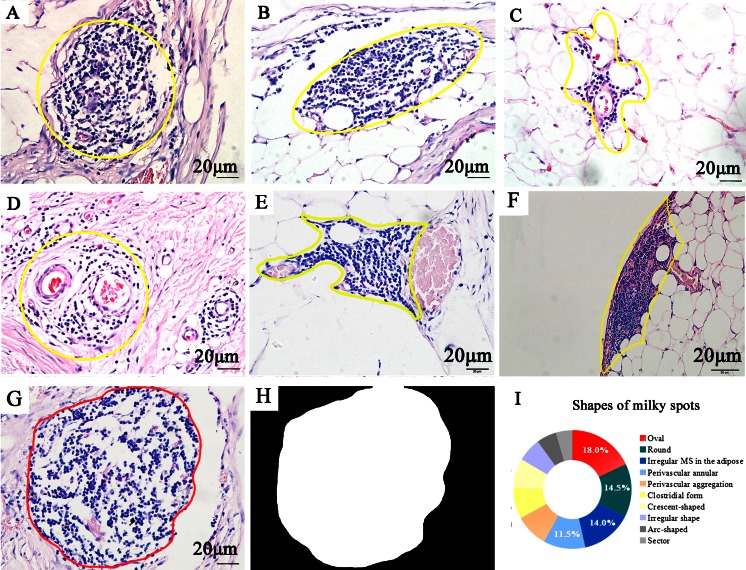
H&E staining of milky spots. The *yellow frame* is used to show the gross morphology of MS. **a** Round. **b** Oval. **c** Irregular form in adipose tissues. **d** Perivascular annular. **e** Perivascular aggregation. **f** Clostridial form. **g**, **h** Computer methods were used to convert HE images into binary images for the calculation of MS perimeter. **i** Proportions of MS shapes (**a**–**h** ×400, *scale bar* = 20 μm)

In our experiments, hematoxylin and eosin (H&E) staining method was manipulated to study 200 MS slides obtained from the omentum specimens of gastric cancer and rectal cancer patients. These slides were examined under Olympus BX51 microscope equipped with an Olympus DP72 camera (Olympus Optical Co., Ltd., Tokyo, Japan) at 100, 200, and ×400 magnifications, and the images were captured by DP72 camera. Then, we drew the outline of every MS images under the guide of expert pathologist. The morphological features of MS were shown as different shapes with corresponding proportions (Fig. 1i). A self-adaptive Otsu threshold method [34] was adopted to convert the obtained images into binary images (Fig. 1g, h). Then, the perimeter of MS region could be output as pixel value spontaneously. Shapes of MS were mainly round, oval, and irregular form in the adipose and perivascular annular (Fig. 1). The median MS perimeter was 2752 (range 817∼7753) computer-based pixels. Considering 125 pixels were equal to 20 μm in these images at ×400 magnification, the median MS diameter of our results was about 140.2 (41.6–395.1) μm.

### Cellular constituents

Recent researches demonstrate that MSs are small specific structures devoid of capsule, formed between 20th and 35th week of gestation [32], consisting of macrophages, lymphocytes, and some plasma cells supplied by blood and lymphatic vessels [35–37].

**Fig. 2:**
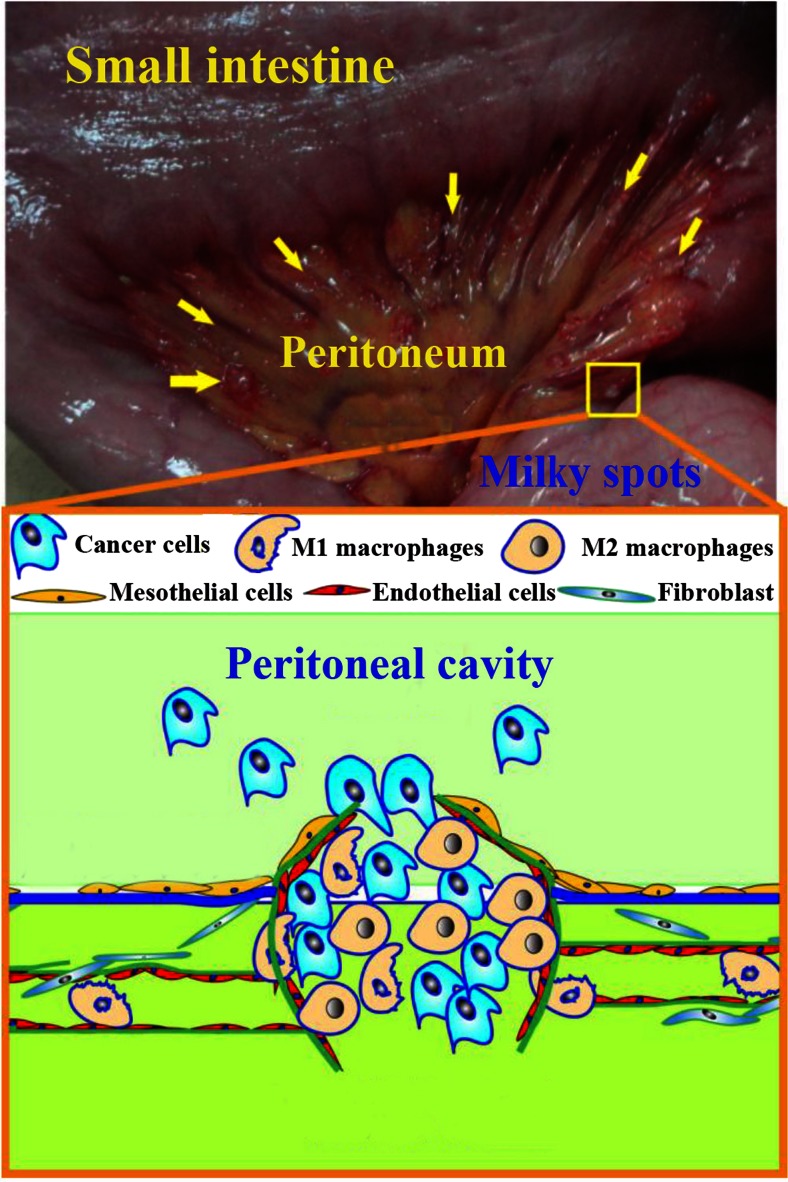
Structure of milky spots. MSs are small specific structures composed of macrophages, lymphocytes, and some plasma cells that aggregate in the perivascular region

In the omentum, abundant immunocytes aggregate in the perivascular region to constitute MS (Fig. 2). At the microscopic level, MSs contain a glomerular-like capillary network of blood vessels which enables fluid exchange between the peritoneal cavity, the blood stream, and the surrounding omental tissue [38, 39]. Diameters of macrophages with irregular shape vary from 15 to 20 μm. Diameters of both B and T lymphocytes vary from 7 to 10 μm [33].

The above cellular compositions are arranged around the omental glomeruli that lie directly beneath the discontinuous mesothelium layer [23], which are characterized by the presence of pores or stomata, allowing for direct communication with the peritoneal cavity [40]. Typically under the scanning electron microscopy, macrophages of activated MS were found to change their membrane activity prominently and migrate through the intercellular stomata of MS mesothelial cells into the peritoneal cavity [41–43].

### Cellular percentage and distribution

**Fig. 3:**
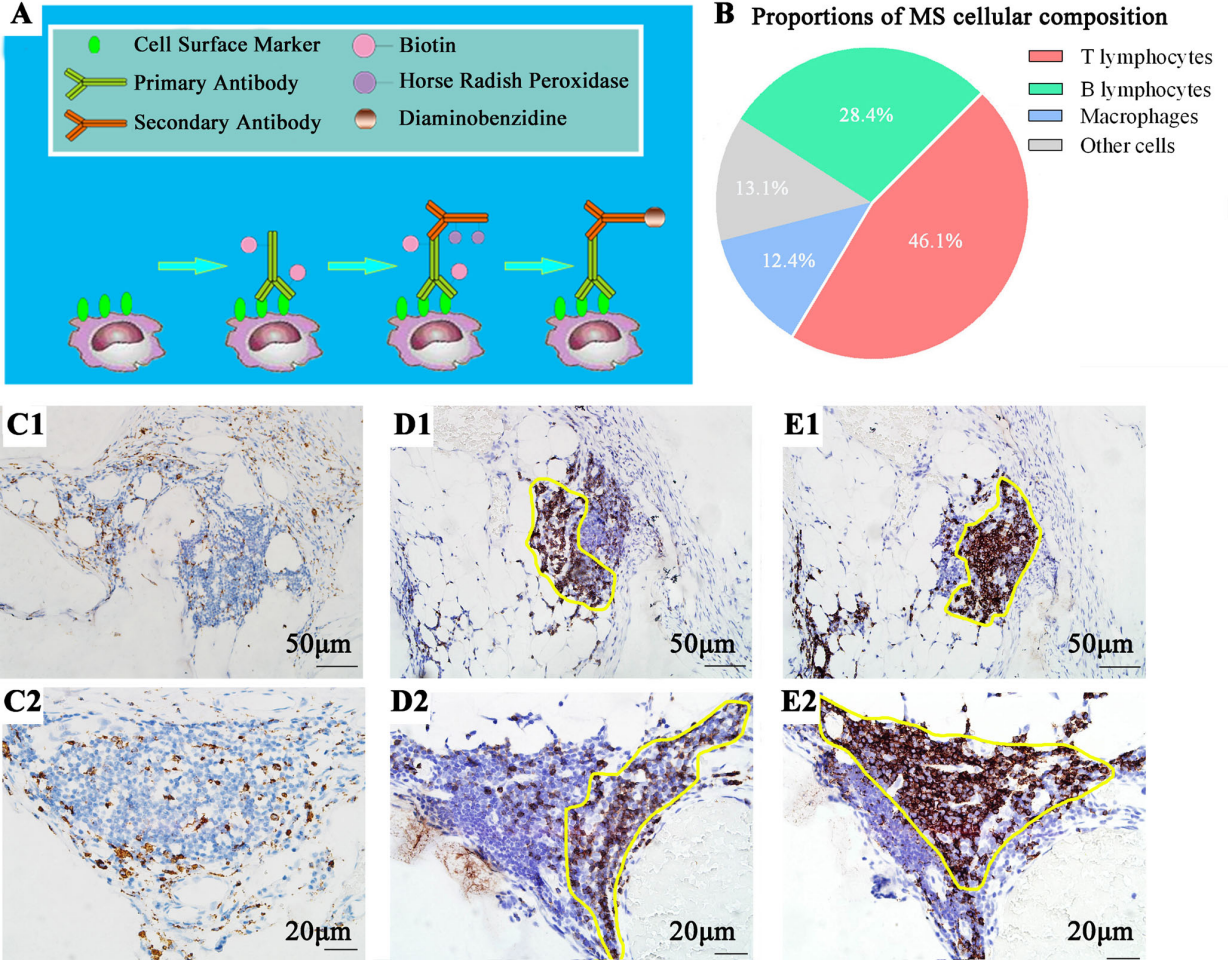
IHC staining of milky spots to analyze the cellular percentages. **a** Procedures of IHC study. **b** Cellular composition of human milky spots. **c** Macrophages are diffusely distributed within MS. **d** + **e** Lymphocytes are located intensively in a particular position. The location of T lymphocytes is roughly complementary with B lymphocytes, as shown by the *yellow frame* (**c1**–**e1** ×200; *scale bar* = 50 μm; **c2**–**e2**; ×400; *scale bar* = 20 μm)

For different experimental subjects and different states of the greater omentum, percentages of MS cellular constituents also differed to some extent, such as macrophages, T lymphocytes, and B lymphocytes (Table 1). As few authors recently had tried to update the cellular knowledge of MS, we conducted immunohistochemical study to quantitatively analyze MS cellular constituents. For the demonstration of macrophages, T lymphocytes and B lymphocytes, the indirect avidin-biotin-peroxidase technique with the monoclonal antibodies PG-M1, F7.2.38, and L26 (DAKO, Denmark) was used. These antibodies distinguish the human leukocyte differentiation antigens CD68, CD3, and CD20cy, respectively. Our results are also shown in Table 1 and Fig. 3.

**Table 1:** Different results of milky spot cellular percentages

Authors	Year	Studying model	States of the omentum	Macrophages	T lymphocytes	B lymphocytes
Beelen H et al. [42]	1988	Male Wistar rats	Cell suspensions; acute inflammatory state	30.0 %	20.0 %	10.0 %
Shimotsuma et al. [31]	1991	8-month-old infants with neuroblastoma	Normal	47.5 %	11.7 %	29.1 %
Krist LF et al. [23]	1995	Male patients	Without intra-abdominal infection or malignancy	67.9 %	10.2 %	10.1 %
Liu et al.	2015	Gastric patients and rectal patients	Normal	12.4 %	46.1 %	28.4 %

Previous researches indicated that macrophages and lymphocytes did not show any preferential pattern of distribution in the MS [23, 33]. However, macrophage precursors were centrally localized inside the MS, while more differentiated cells were found in peripheral areas [24]. Referring to our immunohistochemistry (IHC) studies of MS, macrophages preferentially distributed diffusely, while lymphocytes were located intensively in a particular position, and the areas of T lymphocytes seemed to be roughly complementary with B lymphocytes (Fig. 3b, c).

### Histological staining methods

MS can be observed with the naked eye only by experts in perivascular fatty tissue of the omentum. Therefore, they are more often stained both in vivo and in vitro for easier observation at the microscopic level. Since macrophages in MS can engulf the carbon or ink particles into their cytoplasm, researchers in the beginning injected active carbon [10, 44–46] or black ink [42] into the serous cavities of live animals to facilitate the detection and observation, showing MS as tiny black dots after about 20 min. In addition, macrophages and T lymphocytes located in the milky spots were coloring dark red by nonspecific esterase stain [10].

Conventional H&E and Giemsa methods are also applied to stain MS internal immune cells. Collins et al. [39] showed that the surface mesothelium layer of human MS was loosely arranged without basement membrane, and macrophages were occasionally found among the neighboring mesothelial cells. Capillaries with high density and different levels constituted the basic skeleton of MS within the internal layer. Other H&E staining researches [11, 27, 42, 46, 47] found similar results that these purple cellular clusters stained by hematoxylin constitute MS together with pink blood vessels stained by eosin. However, immune cells could not be differentiated into specific types for further studies only by H&E staining. Therefore, IHC/immunocytochemistry methods [11, 12, 24, 30, 32, 33, 48, 49] were applied using homologous monoclonal antibodies for the different leukocyte superficial differential antigens of MS cellular compositions. On the basis of traditional IHC methods, immunofluorescence staining [30, 50] featured by the fluorescence of GFP-tagged immune cells turned out to be more obvious for the detection of MS macrophages.

To date, extensive biophysical and biochemical staining methods have been used to detect MS (Table 2). However, nearly all researches failed to obtain in situ quantitative information with morphological features for multiple cellular constituents of MS except for Gerber et al. [30], who succeeded in staining simultaneously with anti-CD45 to label immune cells (red), anti-CD31 to mark blood vessels (green), and anti-LYVE-1 (yellow) to identify lymphatic vessels.

**Table 2:** Histological staining methods for observing milky spots

Staining methods	Researches	Year	Studying model	Stained content of MS	Result
Toluidine-blue staining	Shimotsuma et al.[31]	1991	Infants	Mast cells	Purple
Activated carbon	Clark et al. [44]	2013	Mouse	MS, macrophages	Black
	Di Paolo et al. [48]	2005	Mouse		
	Hagiwara et al. [10]	1993	Mouse		
	Shimotsuma et al. [43]	1989	Mouse		
India ink	Krishnan et al. [40]	2012	Mouse	MS, blood vessels	Black
H&E staining	Clark et al. [44]	2013	Mouse	Immune cells	Purple
	Krishnan et al. [40]	2012	Mouse		
	Panasco et al. [25]	2010	Mouse		
	Khan et al. [11]	2010	Mouse		
	Abe et al. [45]	2009	Mouse		
	Collins et al. [37]	2009	Human	MS	Purple cellular clusters
Nonspecific esterase stain	Hagiwara et al. [10]	1993	Mouse	Macrophages, T lymphocytes	Dark red
Giemsa staining	Clark et al. [44]	2013	Mouse	Immune cells	Dark staining areas
	Panasco et al. [25]	2010	Mouse		
Immunohisto-/cyto-chemistry	Sedlacek et al. [46]	2013	Mouse	Immune cells	With reference to the original literature
	Khan et al. [11]	2010	Mouse		
	Sorensen et al. [12]	2009	Mouse		
	Gerber et al. [28]	2006	Mouse		
	Krist et al. [30]	1997	Infants		
	Shimotsuma et al. [31]	1991	Infants		
	Wijffels et al. [24]	1992	Mouse		
	Beelen et al. [42]	1988	Mouse		
Immunofluorescence staining	Oosterling et al. [47]	2006	Mouse	Macrophages	Green
	Gerber et al. [28]	2006	Mouse	GFP-tagged immune cells	Green
Infiltrated tumor cells	Tsujimoto et al. [19]	1996	Mouse	Melanoma cells	Black

*GFP* green fluorescent protein

## Physiological functions of MS

The omentum is characterized by a single layer of mesothelial cells and a submesothelial region composed of connective tissue with a few fibroblasts, mast cells, macrophages, and blood vessels [51]. As the central regulator of peritoneal homeostasis, the omentum performs active functions in regulating fluid and solute transport, sensing and repairing injuries, promoting angiogenesis, fighting infection, providing stem cells, producing regulatory molecules, and storing and supplying lipids [14]. The MS cellular compositions can be classified into structural, migratory, and functional elements. Fibroblasts, adipocytes, mesothelia, and endothelia compose the structural elements. Lymphocytes, granulocytes, and monocytes make up the migratory elements. The functional element is comprised of macrophages, stromal cells, and high endothelium of the veins [52].

### Secondary lymphoid organs in the omentum

As mentioned above, MSs were identified as high densities of immune cells located atop densities of capillaries within the surrounding adipocytes from H&E stained images [11, 27, 39, 42, 47, 49]. A glomerular-like capillary network within MS enables fluid exchange between the peritoneal cavity, the bloodstream, and the surrounding omental tissue [39]. Immunocytes in MS play an important role in peritoneal immunity. Omental MS and macrophages could be activated by i.p. injection of a streptococcal preparation, OK-432 [41]. The activated macrophages demonstrate marked surface membrane activity and migration capability and participate in peritoneal immunoreactions against foreign matters. Ultimately, the structural integrity of MS was partially lost by the injected streptococcal preparation [41] or tumor cells [30].

For long it has been controversial as to whether to regard MS as secondary lymphoid organs for their lack of dendritic cells as well as follicular dendritic cells compared with conventional lymphoid organs. However, compelling data provided by Rangel-Moreno et al. [25] terminated the debated the classification above. Using splenectomized lymphotoxin-alpha-deficient mice (*Lta* ^*-/-*^), which already lacked lymph nodes and Peyer’s patches, they creatively designed SLP mice model by reconstituting *Lta* ^*-/-*^ with wild-type bone marrow. As summarized by Mebius [26] later, antigens injected into the peritoneal cavity of SLP mice were shown to accumulate in MS, resulting in the generation of antigen-specific antibodies. Germinal center B cell responses and proliferation of T cells in response to intraperitoneally injected antigens could also be observed in MS. Although develop in the absence of lymphoid tissue inducer cells, MSs function as unique secondary lymphoid organs that promote immunity to peritoneal antigens.

### Suppliers of peritoneal macrophages

MSs are also considered as the site for the generation and differentiation of peritoneal macrophages [24, 41, 53–55]. Based on ultrastructural endogenous peroxidase cytochemistry using a panel of monoclonal antibodies that recognize precursor cells antigens in the MS, the precursors of macrophages were identified as from the mononuclear phagocyte system (MPS) [24]. As precursor cells differentiated, developing MS contained macrophages in the different stages of maturation during the 20th to 35th weeks of gestation [32]. These peritoneal macrophages participate in the absorption and clearance of bacteria and debris from the peritoneal cavity [41] and act as the frontier line of immune defense along with MS surface mesothelium.

## Pathological reaction of MS

### Inflammatory reaction

Although the omentum is not inherently motile, experimental models have shown that, in response to foreign matters or inflammation, omental blood flow increases, and the proportion of MS expands. In inflammatory conditions, MS can provide immunocytes expressing stem cell markers [56, 57] as well as inflammatory, hemostatic, and chemotactic substances [58] such as vascular endothelial growth factor (VEGF) and basic fibroblast growth factor (bFGF) [59]. These activated cells accumulate to injured sites [60] and promote the recruitment of inflammatory cells [41] within the peritoneal cavity, thus speeding tissue repair and regeneration. During the delayed reaction period, macrophage cytoplasms increase and travel through the MS stomata into the peritoneal cavity to limit inflammation [41, 43].

### Insufficient antitumor effect of MS

#### Dual-functioning roles of tumor-associated macrophages in MS

On the one hand, omental MSs are cytotoxic against PECC to show tumor-resistant effect; on the other hand, they become a highly efficient “natural filter” for screening cancer stem cells [13, 61], thus providing a microenvironment in which cancer cells niche in this region to form a metastatic foci [12, 31, 62]. This phenomenon owes largely to the distinct roles of tumor-associated macrophages, which respond to the presence of stimuli in the different parts of tumors with the release of a distinct repertoire of growth factors, cytokines, chemokines, and enzymes that regulate tumor growth, angiogenesis, invasion, and metastasis [63].

Throughout the life span of PECC within MS, from early-stage tumor micro-metastatic foci that are beginning to vascularize to late-stage tumors that PC occurs, monocytes migrate through blood vessels into MS region under the influence of tumor-derived chemoattractants, including colony-stimulating factor-1 (CSF-1/M-CSF), the CC chemokines, and VEGF [64]. Then they continually proliferate and differentiate into tumor-associated macrophages (TAMs), which typically have been categorized by the dichotomy of classical (M1) and alternative (M2) statuses [65]. M1 are described as pro-inflammatory with tumor-resistant effects. In contrast, M2 are associated with immunosuppression, modifications of extracellular matrix (ECM), and promotion of tumor angiogenesis and metastasis [66–68]. During tumor progression, TAMs have a remarkable degree of plasticity for undergoing phenotypic switch from M1 to M2 [69]. In different microenvironments, TAMs act distinctly to promote cancer cell motility in the areas of invasion, to promote metastasis in stromal and perivascular areas, and to stimulate angiogenesis in avascular and perinecrotic hypoxic areas [63].

Peritoneal mesothelial cells and MS macrophages constitute the first line of defense in the peritoneum. However, PECC can attach to the peritoneum either by the surface of mesothelial cells or the exposed ECM. The attachment of PECC to mesothelial monolayers is mediated by different adhesion molecules like intercellular cell adhesion molecule-1 (ICAM-1) and vascular cell adhesion molecule 1 (VCAM-1) [70–72]. Attachment of PECC to the ECM is mainly mediated by integrin [73, 74]. On one hand, the structure and function of mesothelial cells play roles in resisting the adhesion and colonization of PECC [75]. On the other hand, mesothelial cells possess both epithelial and mesenchymal characteristics and readily undergo epithelial-mesenchymal transformation (EMT) and myofibroblast transformation in response to apoptosis and fibrosis induced by both PECC and TAMs [76, 77]. A TAM-induced HPMC injury model [77] indicated that TAMs directly enhance the invasive ability of PECC and further degrade the integrity of mesothelial cells, enhancing the opportunity for PECC adhesion and colonization. Once injured, the adjacent mesothelial cells will be changed into a cuboidal morphology, and the underlying ECM will be exposed to PECC invasion. The fibroblasts are probably the most abhorrent elements of ECM. Activated by TGFβ-1 signaling, cancer-associated fibroblasts (CAFs) play roles in promoting cancer growth, adhesion, and invasion [78–80]. As collaborative assistants and along with mesothelial cells and ECM, MS TAMs can provide a favorable environment for PECC invasion.

#### Milky spots: a hypoxic niche for peritoneal cancer

It is widely accepted that the growth and spread of cancer cells require angiogenesis, a process by which neo-vessels sprout from the existing vasculature. There are hypoxic areas in metastatic foci of MS because neo-vessels are disorganized and prone to collapse. TAMs accumulate in such hypoxic areas and upregulate VEGF and other proangiogenic factors [81], such as TNF-a, IL-8, and bFGF. Additionally, TAMs also synthesize the elevated levels of MMP-7 [82], which can stimulate endothelial cell proliferation and migration to support tumor angiogenesis [83], thus providing energy for tumor cell proliferation.

Recent researchers have also observed a positive correlation between hypoxic microenvironment and PC and revealed hypoxia-inducible factor-1α (HIF-1α) expression in MS as a predominant factor [84] that controls the tumor stem cell phenotype. As mentioned above, MS could only eliminate mature tumor cells rather than tumor stem/progenitor cells [13, 61]. As a subunit of HIFs regulated by oxygen levels, HIF-1α can trigger a set of adaptive transcriptional responses to regulate tumor stem cell differentiation and self-renewal [85, 86]. On one hand, stem cell-related protein expression is correlated with HIF-1α overexpression. Tumor stem cells’ self-renew is enhanced by hypoxia through HIF-1α, but their differentiation ability is reduced [84], thus leading to the accumulations of abundant tumor stem cells in MS hypoxic areas and ultimately promoting a more aggressive tumor phenotype. On the other hand, epithelial-mesenchymal transition could be induced by hypoxia through HIF-1α, thus enhancing PC occurrence ratio and indicating a negative prognosis for cancer patients.

#### Tumor promotion effect of adipocytes surrounding MS

As is known, the majority of the omentum is composed of bands of adipose tissue that mainly contain adipocytes [87]. In general, the well-known functions of adipocytes include lipid storage, production of endocrine molecules [88, 89], and serving as an integrating hub for inflammation, metabolism, and immunity [46]. However, the roles of adipocytes to promote homing, migration, and invasion of cancer cells were first reported by Nieman et al. [90]. In their co-culture model of ovarian cancer cells and adipocytes, the lipolysis of adipocytes and β-oxidation in cancer cells indicated that adipocytes could act as an energy source for cancer cells. Clark et al. [46] developed this finding into a two-step model for omental colonization mediated by both omental MS and adipocytes. The first step for the adhesion and localization of PECC is dependent on MS. Then adipocytes provide fatty acids for rapid proliferation and tumor growth, thus promoting subsequent spread of PECC to other sites. Therefore, MS and adipocytes play distinct and complementary roles in PECC metastatic colonization within peritoneal cavity.

#### Relationships between MS and PC

Primary tumors of the omentum are rare; however, it is the most favorite site of peritoneal metastasis for cancers of the stomach, colorectum, and ovary. Invasive cancer cells exfoliate from primary tumors, transfer through peritoneal fluid or ascites [91, 92], and preferentially attach the peritoneum, predominantly the greater omentum. Some differences do exist in the pattern or mechanism of MS-related PC from different primary tumors. The omentum is a fatty tissue that connects the spleen, stomach, pancreas, and colon [93]. Owing to positional adjacency to the omentum and the existence of gravity, the anatomical particularity of stomach/colon makes it much easier for cancer cells from gastrointestinal system to undergo implantation metastasis. Except for the above straightway metastatic route, gastric cancer cells and colorectal cancer cells could also be transported by peritoneal fluid [94]. However, ovarian cancer cells have access to and can potentially lodge within the omental MS only through the peritoneal fluid [95]. Although PECCs from different origins demonstrate diversities during the early step of peritoneal metastasis, PECCs have all experienced the following aspects in the process: adhesion, degradation, migration, angiogenesis, and immune evasion [7–9].

The peritoneum is comprised of a single layer of mesothelial cells and its associated underlying ECM [96]. The mesothelium is inevitably damaged after interactions with PECC, particularly for PC patients having surgical trauma and stress [97]. Then, exposed MSs lying beneath the mesothelium actively take over the mission to fight against PECC. The MS-driven model attributing to the peritoneal dissemination of different tumor cells (melanoma cells, ovarian cancer cells, colon cancer cells, etc.) is based on a large body of in vivo data showing that tumor cells rapidly and specifically attach, invade, and proliferate within MS after intraperitoneal injection [12, 30, 42, 46, 49, 50, 61]. For example, ovarian cancer cells rapidly localize to the mice omentum within minutes after intraperitoneal injection [10, 11, 28]. For different experimental models or detection methods, the earliest occurrence time of PECC infiltration within MS varies from hours [50, 61] to days [30, 42]. However, infiltrated PECCs from gastrointestinal or gynecological cancer undergo similar temporal dynamics before apparent tumor nodules proliferate in the omentum. Herein, cancer immunoediting theory by Duun GP et al. [9] is recommended to explain the three stages of interplay between PECCs and MSs. In the beginning, both sides are influenced by each other and to some extent well prepared for the “battle” of occupying MS areas. In the second stage, the number of total immune cells in MS increases in accordance with the visual enlargement of MS size. Mainly due to the cytotoxic abilities of macrophages mentioned above, numbers of PECC decline gradually prior to next stage. However, the screened cancer stem/progenitor cells retain the potency to proliferate and differentiate after the apoptosis of mature cancer cells [13, 61]. Ultimately, proliferating cancer cells in MS areas form micro-metastasis, whereas the structure of MS is disrupted leaving segmented even sporadic immune cells within the tumor mass [30].

**Fig. 4:**
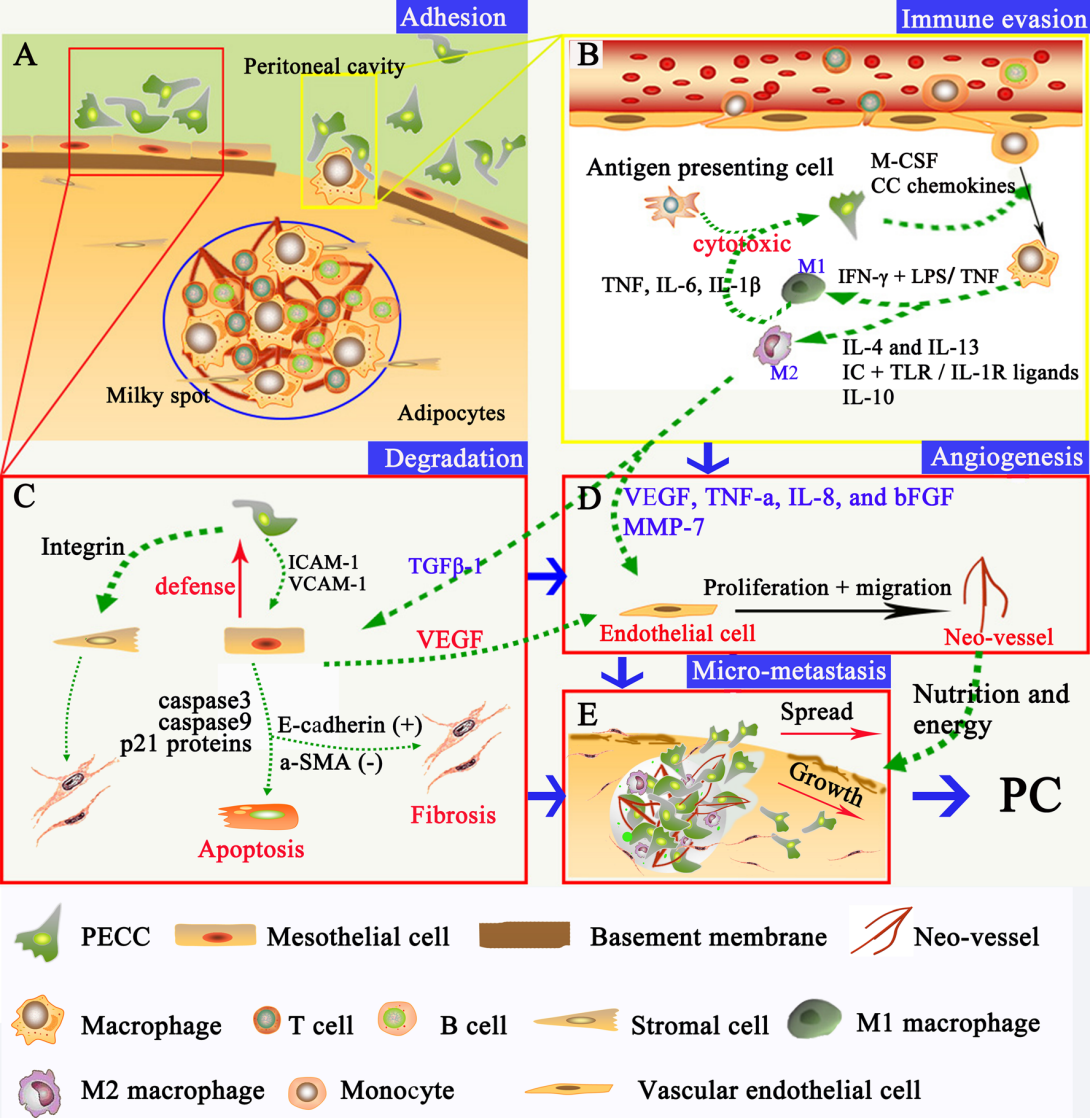
Interactions between PECC and MS cellular constituents. **a** Adhesion of PECC onto mesothelial cells or through the MS stomata. **b** Dual-functioning roles of TAMs in MS. Monocytes migrate through the blood vessels into MS region under the influence of tumor-derived chemokines and continually differentiate into TAMs, including M1 and M2 macrophages. M1 are pro-inflammatory with tumor-inhibiting effects, while M2 favor tumor growth and metastasis. **c** Apoptosis and fibrosis of mesothelial cells by both PECC and M2 macrophages. **d** Tumor angiogenesis within MS. VEGF is the most important factor to promote proliferation and migration of endothelial cells. **e** Formation of micro-metastasis within MS before PC occurs

Therefore, resident immune cells in MS are not able to prevent tumor growth [28, 50]; instead, pro-inflammatory cytokines secreted from cancer, stromal, mesothelial, and immune cells, particularly macrophages, contribute to an inflammatory environment that promotes peritoneal metastasis [98–100]. Furthermore, a large number of adipocytes surrounding MS may promote the growth of the attaching cancer cells by providing lipids to meet their energy demands [90]. The impact of MS on peritoneal metastasis is profound and transforms the initial pattern of micro-metastatic foci into a widespread peritoneal carcinomatosis [101, 102]. Based on current understanding, the involvement of MS in peritoneal carcinomatosis formation could be summarized in Fig. 4.

## Conclusions

With expanding knowledge in the fields of MS morphological and functional studies, these tiny functional units located predominantly within human omentum have been turned out to be hotbeds for peritoneal exfoliated cancer cells. In-depth knowledge and renewing concepts of MS contribute to understanding the multistage development of PC.

## Abbreviations

PC: Peritoneal carcinomatosis
PECCs: Peritoneal exfoliated cancer cells
MSs: Milky spots
GC: Gastric cancer
IVIS: In vivo imaging system
GFP: Green fluorescent protein
H&E: Hematoxylin and eosin
BSA: Bovine serum albumin
IHC: Immunohistochemistry
MPS: Mononuclear phagocyte system
VEGF: Vascular endothelial growth factor
bFGF: Basic fibroblast growth factor
CSF-1: Colony-stimulating factor-1
TAMs: Tumor-associated macrophages
ECM: Extracellular matrix
ICAM-1: Intercellular cell adhesion molecule-1
VCAM-1: Vascular cell adhesion molecule 1
EMT: Epithelial-mesenchymal transformation
CAFs: Cancer-associated fibroblasts
HIF-1α: Hypoxia-inducible factors-1α
